# Effect of titanium dioxide nanotubes in Ca(OH)_2_-based intracanal medicaments on odontoblast-like cells

**DOI:** 10.1590/1807-3107bor-2026.vol40.002

**Published:** 2026-03-06

**Authors:** Matheus Araújo Brito Santos Lopes, Bruna Carolina Costa, Francisco Humberto Nociti, Paulo Noronha Lisboa, Kamila Rosamilia Kantovitz

**Affiliations:** (a) Faculdade São Leopoldo Mandic, SLMANDIC, Dental Material Area, Campinas, SP, Brazil.; (b) Department of Physics, School of Science, São Paulo State University, UNESP, Bauru, SP, Brazil.; (c) American Dental Association Science and Research Institute, ADASRI, Director, Cellular and Molecular Biology Research Group, Acting Senior Director, Innovation and Technology Research, Gaithersburg, MD, USA.; (d) Division of General Practice, Department of Comprehensive Dentistry, University of Maryland, School of Dentistry, UMBSOD, Baltimore, MD, USA.

**Keywords:** Calcium hydroxide, Nanotubes, Titanium

## Abstract

This study investigated the effects of incorporating 3wt% titanium dioxide nanotubes (nTiO_2_) into calcium hydroxide [Ca(OH)_2_]-based intracanal medicament (ICM) formulations on the proliferation, viability, and mitochondrial metabolic activity of mouse dental papilla cells (MDPC-23). Five experimental groups were evaluated (n=3 per group): Cells (control), Calen, Calen + 3wt% nTiO_2_, Ultracal, and Ultracal + 3wt% nTiO_2_. Cell proliferation, viability, and mitochondrial metabolic activity were assessed using the Trypan Blue exclusion and MTT [3-(4,5-dimethylthiazol-2-yl)-2,5-diphenyltetrazolium bromide] assays at 24, 48, and 72 h. Data analysis included the Shapiro–Wilk and Levene tests and generalized linear models (**p**<0.05). At 24 and 48 h, both Ultracal and Ultracal + 3wt% nTiO_2_ showed significantly higher proliferation than the Calen groups (*p*<0.0001). The control group (Cells) exhibited the highest viable cell counts (*p*<0.0005). At 72 h, Calen + 3wt% nTiO_2_ showed the lowest proliferation compared with both the Cells and the Ultracal groups (*p*<0.0001). No significant differences were observed in non-viable cell counts or viable/non-viable ratios over time (p>0.05). Furthermore, Calen and Calen + 3 wt% nTiO_2_ at 24 and 48 h, as well as Calen + 3wt% nTiO_2_ at 72 h, demonstrated the lowest metabolic activity (p<0.0001), with no significant effect of nTiO_2_ (*p*>0.05). No significant intra-group differences over time were observed for the Cells or Ultracal groups (*p*=0.0608 and *p*=0.1417, respectively). Overall, the incorporation of nTiO_2_ did not adversely affect the biological parameters of Ca(OH)_2_-based ICMs assessed in this study. Further investigations are warranted to elucidate potential clinical implications.

## Introduction

The American Association of Endodontists (AAE) recommends intracanal medicaments (ICMs) containing calcium hydroxide [Ca(OH)_2_] for treating traumatized, infected, or perforated immature permanent teeth.^1^ This recommendation is supported by its established antimicrobial and anti-inflammatory properties, along with its demonstrated ability to promote tissue repair.^2^ These effects are attributed to the release of hydroxyl and calcium ions from the Ca(OH)_2_ paste, which inactivate enzymes necessary for bacterial nutrition and disrupt cytoplasmic membranes.^3^ In addition, Ca(OH)_2_ activates tissue enzymes such as alkaline phosphatase, which supports mineralization and helps prevent root resorption.^4^ Although elevated pH levels have been reported as a key factor in root canal disinfection, failure rates of approximately 20% suggest that Ca(OH)_2_-based ICMs may be unable to maintain the high pH needed to eliminate resistant bacteria, such as *E. faecalis*.^2,5^

Recently, interest has grown regarding the potential antimicrobial properties of nanoscale structures incorporated into dental materials.^6-9^ Among these nanomaterials, titanium dioxide nanotubes (nTiO_2_) (a tubular nanostructure of titanium dioxide – TiO_2_) have gained attention due to their high surface-to-mass ratio and enhanced chemical reactivity.^11^ These characteristics facilitate effective chemical interactions with bacteria, aiding in infection control, although potential cytotoxic effects have also been noted.^11,12^ A recent study by our group demonstrated the potential antimicrobial activity of nTiO_2_ incorporated into conventional glass ionomer cement (GIC).^8^ It was found that nTiO_2_ affected S. mutans viability and the expression of key genes essential for bacterial survival and growth, thereby enhancing the anticariogenic properties of GIC.^8^ In contrast, no significant antimicrobial effect was observed when nTiO_2_ was incorporated into a glaze-coated ceramic.^9^ Although several studies have shown that nTiO_2_ may influence the mechanical, physical, and antimicrobial properties of restorative dental materials, limited information is available regarding its potential benefits in endodontic therapy,^6,7,10^ particularly in cases involving bacterial resistance to conventional treatments. In this context, this study aimed to evaluate the effect of incorporating nTiO_2_ into Ca(OH)_2_-based ICMs on the metabolic and proliferative behavior of a pre-odontoblastic cell line (MDPC-23). The null hypotheses were that incorporating 3 wt% nTiO_2_ into Ca(OH)_2_-based ICM would not affect either (a) proliferation and viability or (b) mitochondrial metabolic activity in MDPC-23 cells.

## Methodology

**Figure 1:**
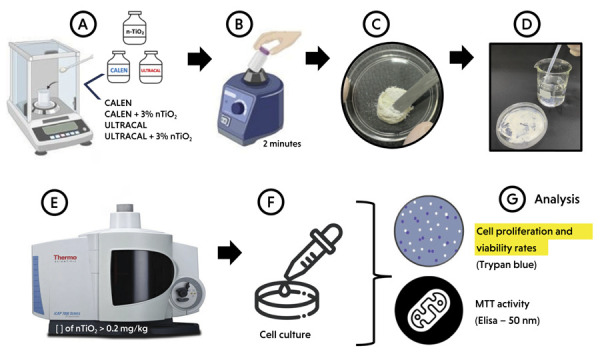
Schematic figure illustrating the methodology used: (a) determining nTiO2 and Ca(OH)2-Based Intracanal Medicaments (ICM) on an accurate scale; (b) adding 3% of nTiO2 into Ca(OH)2-Based ICM by vortexing; (c) Spreading 3 g of Ca(OH)2-based ICM (Calen or Ultracal) with or without nTiO2 to form a thin film; (d) Adding 15 mL of mili-Q water to each plate and keeping it in contact with the ICM film for a period of 72 h; (e) samples were analyzed by optical emission spectrometry with inductively coupled plasma; (f) Cells were seeded and maintained in control or experimental culture medium and (g) the analysis included cell proliferation and viability rates (trypan blue; n=3; at 24, 48, and 72 h) and mitochondrial metabolic activity rate of MDPC-23 cells (MTT; n=3; at 24, 48, and 72 h).

This study evaluated the effects of incorporating nTiO_2_ into a Ca(OH)_2_-based ICM formulation on cell proliferation, viability, and mitochondrial metabolic activity of MDPC-23 cells. Five experimental groups were established (n = 3 per group): (1) Cells (control); (2) Calen; (3) Calen + 3 wt% nTiO_2_; (4) Ultracal; and (5) Ultracal + 3 wt% nTiO_2_. Cell proliferation and viability were determined using the Trypan Blue exclusion assay, which measures bothtotal and non-viable cells. Mitochondrial metabolic activity was assessed by the MTT assay [3-(4,5-dimethylthiazol-2-yl)-2,5-diphenyltetrazolium bromide]. All assays were conducted at 24, 48, and 72 h (Figure 1). The MDPC-23 cell line was originally provided by the Nociti Laboratory (Department of Prosthodontics and Periodontics, State University of Campinas, Brazil) and was available at the Faculdade São Leopoldo Mandic. The experimental protocol was approved by the São Leopoldo Mandic Institutional Animal Care and Use Committee (IACUC; Protocol No. 2023-0028).

## Materials and Methods

The Ca(OH)_2_-based ICMs tested in this study, along with their respective brands, manufacturers, compositions, and batch numbers, are listed in Table 1. The nanotubes (length ~20 nm; diameter ~10 nm) were synthesized by the alkaline method and composed of a single spiral-wound TiO_2_ sheet.^13^ Specifically, the nanotubes were prepared by mixing 12 g of TiO_2_ in anatase form (Aldrich, 99%) into 200 mL of 10 M sodium hydroxide (NaOH). This mixture was placed in an open Teflon container in a glycerin bath maintained at 120 °C for 24 h. The synthesis was conducted at ambient pressure, ensuring that only the precursor reagents were exposed to the alkaline treatment. Following this step, the resulting mixture was thoroughly washed with 0.1 M hydrochloric acid and deionized water to remove residual sodium ions and to adjust the pH to 7. The synthesized nanotubes were then dried in a conventional oven at 200 °C for 24 h under atmospheric conditions.^13^

**Table 1:** Brand, manufacturer, batch and composition of the Ca(OH)_2_-based ICM tested in this study.

Brand	Manufacturer	Batch	Composition
Calen	SS White/BR, Rio de Janeiro, Brazil	#0180821	50% calcium hydroxide, 17% zinc oxide, 3% rosin, 6% paramonochlorophenol, and 24% polyethylene glycol
Ultracal XS	Ultradent Products Inc., Indaiatuba, SP, Brazil	#D0185	35% calcium hydroxide, 20% barium sulphate, 45% water and methylcellulose

Source: Material Safety Data Sheets (MSDS). Available at: https://chemicalsafety.com/sds-search/ (Accessed: 01/20/2024)

The nTiO_2_ was weighed on a precision balance (Adventurer Ohaus, Parsippany, NJ, USA) and manually incorporated into the Ca(OH)_2_-based ICMs (Calen or Ultracal) at a concentration of 3 wt%. The mixture was homogenized in a vortex mixer (QL-901, Biomixer, Taft, CA, USA) for 2 minutes.^8^ This concentration was selected as the maximum amount of nTiO_2_ that could be successfully incorporated while preserving a paste consistency suitable for application and spreading in a Petri dish (Figure 1).^7^

## Determining the Concentration of Titanium in Aqueous Extracts

To assess the release of titanium into the medium over time, 3 g of Ca(OH)_2_-based ICM (Calen or Ultracal) containing 3 wt% nTiO_2_ was spread to form a thin film. Then, 15 mL of Milli-Q water was added to each plate and kept in contact with the ICM film for 24, 48, and 72 h. At each time point, 5 mL of supernatant was collected to determine the concentration of released titanium. The collected samples were digested using nitric acid and hydrochloric acid, and titanium content was measured by inductively coupled plasma optical emission spectrometry (PlasmaQuant PQ 9000 Elite, Analytik Jena) (Figure 1). The 72 h protocol was selected for further analysis, since it exhibited greater data separation among the measured values, making the analysis clearer and more interpretable.

## Biological Evaluation

### Experimental Culture Medium Preparation

Experimental culture media were prepared by incubating 3 g of each ICM formulation—Ca(OH)_2_- and CHX-based ICMs, with or without 3 wt% nTiO_2_—in 20 mL of Dulbecco’s Modified Eagle Medium (DMEM) at 37 °C for 72 h. After incubation, the DMEM was collected and filtered through a 0.22 μm membrane to obtain sterile experimental media for cell culture. DMEM without prior exposure to medication was used as the control.

### Cell Proliferation and Viability Assay - Trypan Blue (n = 3 per group)

MDPC-23 cells were seeded onto sterile 48-well plates (Corning Costar, Cat #CLS3548) at a density of 1 × 10^3^ cells/well and maintained at 37 °C in a humidified atmosphere containing 5% CO_2_ in DMEM supplemented with 10% fetal bovine serum (FBS) (Gibco, Thermo Fisher Scientific, Waltham, MA, USA) for 24 h. After this initial incubation, the culture medium was replaced with either control or experimental medium containing 2% FBS. The viable and non-viable cell counts were assessed at 24, 48, and 72 h using the Trypan Blue exclusion assay and a hemocytometer. Briefly, the cells were trypsinized, centrifuged, and the resulting pellet was resuspended in 1 mL of phosphate-buffered saline (PBS). Then, 20 μL of the cell suspension was mixed with 20 μL of 0.4% Trypan Blue and incubated for 5 minutes prior to counting with a hemocytometer (Sigma-Aldrich, São Paulo, Brazil) (Figure 1). Cells at passages 13 and 14 were used, and all experiments were performed in triplicate and repeated twice to ensure data consistency.

### Mitochondrial Metabolic Activity – MTT Assay (n = 3 per group)

Cells were cultured as described above (Cell Proliferation and Viability Assay) and subjected to the MTT assay at 24, 48, and 72 h to evaluate mitochondrial succinate dehydrogenase activity, which reduces the MTT substrate [3-(4,5-dimethylthiazol-2-yl)-2,5-diphenyltetrazolium bromide] to formazan crystals in viable MDPC-23 cells. Notably, the MTT and proliferation assays were performed simultaneously using aliquots of control and experimental media prepared in a single batch. After removal of the culture medium, each well received 900 μL of DMEM and 100 μL of MTT (5 mg/mL) (Vida Technologies, Cat #M6494, Kalyan, Maharashtra, India) in combination with Dulbecco’s Phosphate Buffered Saline (DPBS) (Gibco). The plates were incubated at 37 °C in 5% CO_2_ for 4 h, protected from light. After incubation, 500 μL of dimethyl sulfoxide (DMSO) (Sigma-Aldrich, São Paulo, Brazil) was added to dissolve formazan crystals formed in viable cell mitochondria. Three 100 μL aliquots from each well were transferred to a 96-well plate (Corning Costar, Corning, NY, USA) and absorbance was measured at 590 nm using an ELISA microplate reader (VersaMax, Molecular Devices, Sunnyvale, CA, USA) (Figure 1).^14^ The mean of the three readings per well was used as the representative value for the respective well in the 48-well plate.

## Statistical Analysis

The normality and homogeneity of variance were assessed using the Shapiro–Wilk and Levene tests (p ≤ 0.05). Generalized linear models were applied to evaluate the effects of nTiO_2_ and time on MDPC-23 cell proliferation and mitochondrial metabolism (α = 0.05). All statistical analyses were conducted using R software [R Core Team (2023). R: A language and environment for statistical computing. R Foundation for Statistical Computing, Vienna, Austria].

## Results

The concentration of nTiO_2_ in the aqueous extracts was determined using inductively coupled plasma optical emission spectrometry. The results showed that titanium concentrations in the experimental medium consistently exceeded 0.2 mg/kg.

**Figure 2:**
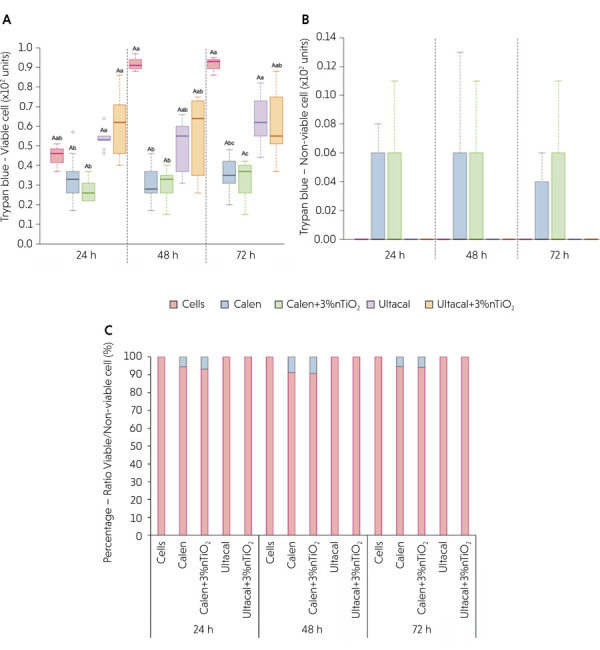
Cell proliferation and viability assay using Trypan blue according to the groups: (A) Box-plot (median, maximum and minimum values) of viable cell counts (in units); (B) Average and standard deviation of non-viable cell counts (in units); (C) the ratio of viable and non-viable MDPC-23 cell counts (in %).

Figure 2 illustrates cell proliferation, including viable and non-viable cell counts and their ratios (expressed as percentages) over time. At 24 h, proliferation was significantly higher in the Ultracal and Ultracal + 3 wt% nTiO_2_ groups compared with the Calen and Calen + 3 wt% nTiO_2_ groups (*p <* 0.0001). After 48 h, viable cell counts in the Calen and Calen + 3 wt% nTiO_2_ groups were lower than those in the Cells group (p < 0.0005). At 72 h, the Calen + 3 wt% nTiO_2_ group exhibited the lowest proliferation compared with both the Cells and Ultracal groups (p < 0.0001). No significant differences were found in non-viable cell counts (Figure 2B) or in the ratio of viable to non-viable MDPC-23 counts over time (p > 0.05) (Figure 2C).

Different capital letters represent statistically significant differences within the same group over time by generalized linear models, while different lowercase letters indicate statistically significant differences between groups over time, at 24, 48, or 72 h (p ≤ 0.05). There was no significant difference in non-viable cells counts or in the ratio of viable and non-viable MDPC-23 cell counts among the experimental groups over time (p≥0.05).

**Figure 3:**
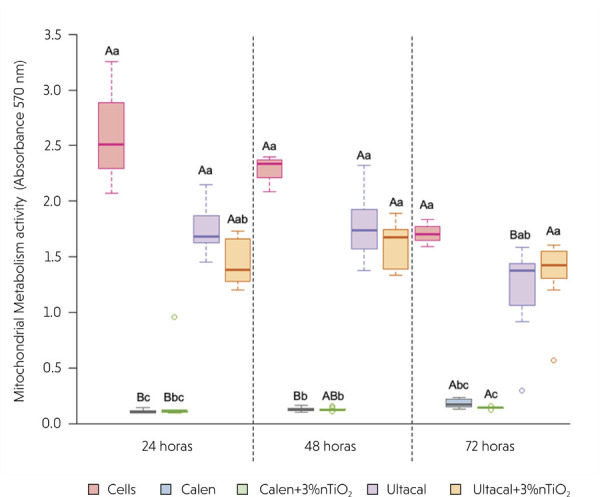
Box-plot (median, maximum and minimum values) of MTT assay (absorbance - 570 nm). Different capital letters represent statistically significant differences within the same group over time by generalized linear models, while different lowercase letters indicate statistically significant differences between groups over time, at 24, 48, or 72 h (p≤0.05).

Figure 3 presents the median values (minimum and maximum) of mitochondrial activity, assessed by the MTT assay, over time. At 24 and 48 h, the Calen and Calen + 3 wt% nTiO_2_ groups exhibited the lowest mitochondrial activity (p < 0.0001). In contrast, the Cells group demonstrated the highest mitochondrial activity (p < 0.0001), and the incorporation of nTiO_2_ into ICMs had no statistically significant effect (p > 0.05). At 72 h, the Calen + 3 wt% nTiO_2_ group again showed the lowest mitochondrial activity (*p <* 0.0001). Intra-group analyses over time revealed no significant difference for the Cells and Ultracal + 3 wt% nTiO_2_ groups (p = 0.0608 and p = 0.1417, respectively). However, both Calen groups—with or without nTiO_2_—exhibited increased mitochondrial activity over time (*p* = 0.0003 and p = 0.0041, respectively).

## Discussion

In the present study, the null hypotheses—that incorporating 3 wt% nTiO_2_ into Ca(OH)_2_-based ICMs does not affect cell proliferation, viability, or mitochondrial metabolic activity—were not rejected (Figure 2 and 3). The incorporation of 3 wt% nTiO_2_ into either Ultracal or Calen resulted in no adverse effect on the proliferation, viability, or mitochondrial metabolic activity (MTT) of MDPC-23 cells over time when compared with their respective formulations without nanotechnology. These findings suggest that the nanomodified ICMs were well tolerated by MDPC-23 cells, which exhibit an odontoblast-like phenotype and play a key role in dentin repair and the preservation of dental tissue integrity, particularly under conditions such as those created by biomechanical root canal preparation.

Although the mechanisms by which metallic nanostructures such as nTiO_2_ interact with cells are not yet fully elucidated, the biocompatibility observed in this study may be attributed to specific psychochemical properties of nTiO_2_, including its surface reactivity, particle size, and interaction with cellular pathways.^15,16^ In the current study, the elevated pH resulting from the release of Ca(OH)_2_ from the ICMs containing nanotechnology may have influenced the formation of reactive oxygen species (ROS) by MDPC-23 cells. TiO_2_ has been shown to affect lipid peroxidation of the cell membrane through electrostatic interactions.^15^ In addition, it may alter cellular permeability by binding to intracellular organelles and biological macromolecules.^15,16^ In the present investigation, no significant effect of nTiO_2_ was observed on any of the biological parameters assessed. These results align with findings from previous studies that evaluated the incorporation of metal nanoparticles into mineral trioxide aggregate (MTA) or Portland cement (at 1 wt%) and demonstrated biocompatibility with fibroblasts.^10,17^ Furthermore, Shahi et al. (2023)^18^ reported that the incorporation of TiO_2_ into MTA and white Portland cement increased their push-out bond strength, suggesting that nTiO_2_ may contribute to the structural reinforcement of materials used in intracanal therapies. These findings support the notion that the structural properties of the material are critical for maintaining the integrity of the treated tooth and, consequently, the viability of surrounding tissue cells. A previous study investigated the antimicrobial effect of ICMs containing nTiO_2_ and found that Calen pastes with incorporated nTiO_2_ exhibited enhanced antimicrobial activity against E. coli.^7^ Ongoing studies in our laboratory aim to further explore the antimicrobial effects of nTiO_2_ when incorporated into Ultracal formulations.

Analysis of cell proliferation and viability rates revealed that nTiO_2_ generally exhibited good biological compatibility, minimizing adverse reactions to surrounding tissues. However, this compatibility appears to be influenced by the composition and concentration of the components in the paste formulation, as well as the duration of exposure to the medium. These findings align with those of a previous study that evaluated the effects of MTA Plus on the viability, apoptosis/necrosis profile, and oxidative stress levels of rat odontoblast-like cells.^20^ That study concluded that cytotoxic effects are both time- and concentration-dependent, and that the risks become negligible once the cytotoxic components have been eluted.^20^

Among the various Ca(OH)_2_-based paste formulations, Ultracal with or without nanotechnology demonstrated a higher number of viable MDPC-23 cells compared with Calen paste, regardless of the incorporation of 3 wt% nTiO_2_ over time (*p <* 0.05). A study by Manaspon et al. (2021)^21^ compared the cellular effects of four Ca(OH)_2_-based dental materials on dental pulp stem cells and provided evidence of the in vitro biocompatibility of ProRoot^®^ MTA and Biodentine™. That study also suggested that these materials, which share a composition similar to that of Ultracal used in the present study, support cellular activities indicative of regenerative potential.

Additionally, a significant increase in mitochondrial metabolic activity was observed at 72 h compared with 24 h, suggesting a time-dependent enhancement of mitochondrial function. This effect may be related to the water solubility of the vehicle used in the Calen paste, specifically polyethylene glycol, which contributes to the gradual and uniform release of disassociated ions. These findings are supported by other studies that examined ion diffusion in Ca(OH)_2_-based ICMs prepared with different vehicles, such as saline solution, anesthetic solution, and polyethylene glycol 400.^22,23^ According to the manufacturer, Ultracal XS^®^ (Ultradent Products) contains 35% Ca(OH)_2_, 20% barium sulfate (radiopacifier), and 45% water and methylcellulose. While the antimicrobial effect of this Ca(OH)_2-_based ICM may be comparable to that of formulations containing up to 50% Ca(OH)_2_,^23^ its influence on mitochondrial activity could lead to a reduction in cellular function after 72 h. This observation suggests that the effectiveness of the ICM as an intracanal dressing may decrease over time, indicating that a shorter interval between clinical sessions may be required. It is important to note that these findings were obtained in a controlled in vitro environment using a single cell line (MDPC-23 cells), which may not fully reflect the complexity of in vivo biological systems. Consequently, the results may not be generalizable to other cell types or to clinical conditions, where variables such as immune responses, tissue interactions, or long-term outcomes could influence the biological behavior of the material.

Future investigations into the incorporation of nTiO_2_ into Ca(OH)_2_-based ICMs should also consider additional psychochemical characteristics, particularly the photocatalytic effects of TiO_2_. The ability of nTiO_2_ to absorb energy and generate free radicals, including hydroxyl radicals, facilitates oxidation reactions and may help reduce the overall toxicity of TiO_2_.^24-26^ Several authors have emphasized the significant role of light in modulating the toxicity of TiO_2_.^25-27^

It is essential to highlight that the methodology employed in this study represents a novel step toward technological innovation aimed at enhancing the biological performance of Ca(OH)_2_-based ICMs. Therefore, further research is required to evaluate MDPC-23 cell proliferation, viability, and mitochondrial metabolic activity using diverse experimental approaches. Such studies will be instrumental in characterizing the cytotoxic, histological, immunological, and physiological effects of these novel strategies compared with those of conventional ICMs.

## Conclusion

In conclusion, the findings indicate that Ultracal significantly enhanced cell viability and mitochondrial metabolic activity compared with Calen. Conversely, Calen maintained mitochondrial activity in MDPC-23 cells, despite being associated with a lower viable cell count. Overall, the results demonstrate that the incorporation of nTiO_2_ did not adversely affect the biological parameters evaluated in this study.

## Data Availability

The authors declare that all data generated or analyzed during this study are included in this published article.

## References

[1] Law AS (2013). Considerations for regeneration procedures. J Endod.

[2] Siqueira JF, Rôças IN (2022). Present status and future directions: Microbiology of endodontic infections. Int Endod J.

[3] Goldberg F, Cantarini C, Alfie D, Macchi RL, Arias A (2020). Relationship between unintentional canal overfilling and the long-term outcome of primary root canal treatments and nonsurgical retreatments: a retrospective radiographic assessment. Int Endod J.

[4] Roig-Soriano X, Souto EB, Elmsmari F, Garcia ML, Espina M, Duran-Sindreu F, Sánchez-López E, González Sánchez JA (2022). Nanoparticles in Endodontics Disinfection: State of the Art. Pharmaceutics.

[5] Jose J, Teja KV, Janani K, Alam MK, Khattak O, Salloum MG, Magar SS, Magar SP, Rajeshkumar S, Palanivelu A, Srivastava KC, Shrivastava D (2022). Preparation of a Novel Nanocomposite and Its Antibacterial Effectiveness against Enterococcus faecalis -An In Vitro Evaluation. Polymers (Basel).

[6] Elmsmari F, Delgado LM, Duran-Sindreu F, Pérez RA, García ML, Teulé Trull M, Afrashtehfar KI, González JA, Sánchez-López E (2023). Novel strategies enhancing endodontic disinfection: Antibacterial biodegradable calcium hydroxide nanoparticles in an ex vivo model. Int J Pharm.

[7] dos-Santos ES, Bridi EC, Basting RT, Nociti FH, Peruzzo DC, França FMG, Pascon FM, Lisboa PN, Kantovitz KR (2024). Do titanium dioxide nanotubes improve the antimicrobial potential and chemical properties of calcium hydroxide pastes?. Dental Press Endod.

[8] Araújo IJS, Ricardo MG, Gomes OP, Giovani PA, Puppin-Rontani J, Pecorari VA, Martinez EF, Napimoga MH, Nociti FH, Puppin-Rontani RM, Lisboa-Filho PN, Kantovitz KR (2021). Titanium dioxide nanotubes added to glass ionomer cements affect S. mutans viability and mechanisms of virulence. Braz Oral Res.

[9] Picolo MZD, Andre CB, Kantovitz KR, Carvalho GLM, Costa BC, Lisboa-Filho PN, Cavalli V (2024). TiO _2_ nanotubes incorporated into a glaze-coating ceramic: surface roughness, color, and antibiofilm activity. Odontology.

[10] Samiei M, Janani M, Asl-Aminabadi N, Ghasemi N, Divband B, Shirazi S, Kafili K (2017). Effect of the TiO_2_ nanoparticles on the selected physical properties of mineral trioxide aggregate. J Clin Exp Dent.

[11] Vimbela GV, Ngo SM, Fraze C, Yang L, Stout DA (2017). Antibacterial properties and toxicity from metallic nanomaterials. Int J Nanomedicine.

[12] Antonopoulou M (2022). Homogeneous and Heterogeneous Photocatalysis for the Treatment of Pharmaceutical Industry Wastewaters: A Review. Toxics.

[13] Arruda LB, Santos CM, Orlandi MO, Schreiner WH, Lisboa-Filho PN (2015). Formation and evolution of TiO _2_ nanotubes in alkaline synthesis. Ceramics International.

[14] Cibim DD, Saito MT, Giovani PA, Borges AFS, Pecorari VGA, Gomes OP, Lisboa-Filho PN, Nociti-Junior FH, Puppin-Rontani RM, Kantovitz KR (2017). Novel Nanotechnology of TiO _2_ Improves Physical-Chemical and Biological Properties of Glass Ionomer Cement. Int J Biomater.

[15] Szymanska R, Kolodziej K, Slesak I, Zimak-Piekarczyk P, Orzechowska A, Gabruk M, Zadlo A, Habina I, Knap W, Burda K, Kruk J (2016). Titanium dioxide nanoparticles (100-1000 mg/l) can affect vitamin E response in Arabidopsis thaliana. Environ Pollut.

[16] Navarro-Requena C, Weaver JD, Clark AY, Clift DA, Pérez-Amodio S, Castaño Ó, Zhou DW, García AJ, Engel E (2018). PEG hydrogel containing calcium-releasing particles and mesenchymal stromal cells promote vessel maturation. Acta Biomater.

[17] Zadsirjan S, Dehkordi NP, Heidari S, Najafi F, Zargar N, Feli M, Salimnezhad S (2024). Synthesis of a Calcium Silicate Cement Containing a Calcinated Strontium Silicate Phase. Int J Dent.

[18] Shahi S, Samiei M, Bahari M, Yavari H, Rahbar Mahvarian M (2023). Effect of Incorporating Titanium Dioxide Nanoparticles into White Portland Cement, White Mineral Trioxide Aggregate, and Calcium Enriched Mixture Cement on the Push-out Bond Strength to Furcal Area Dentin. J Dent (Shiraz).

[19] Neunzehn J, Pötschke S, Hannig C, Wiesmann HP, Weber MT (2017). Odontoblast-like differentiation and mineral formation of pulpsphere derived cells on human root canal dentin in vitro. Head Face Med.

[20] Eid AA, Gosier JL, Primus CM, Hammond BD, Susin LF, Pashley DH, Tay FR (2014). In vitro biocompatibility and oxidative stress profiles of different hydraulic calcium silicate cements. J Endod.

[21] Manaspon C, Jongwannasiri C, Chumprasert S, Sa-Ard-Iam N, Mahanonda R, Pavasant P, Porntaveetus T, Osathanon T (2021). Human dental pulp stem cell responses to different dental pulp capping materials. BMC Oral Health.

[22] Poorni S, Miglani R, Srinivasan MR, Indira R (2009). Comparative evaluation of the surface tension and the pH of calcium hydroxide mixed with five different vehicles: an in vitro study. Indian J Dent Res.

[23] Kibe AN, Nikhade PP, Thote AP (2023). Comparative Evaluation of the Effect of Six Different Low-Surface-Tension Vehicles on the Penetration of Modified Triple Antibiotic Paste in Dentinal Tubules: An In Vitro Study. Cureus.

[24] Wang J, Du L, Fu Y, Jiang P, Wang X (2019). ZnO nanoparticles inhibit the activity of Porphyromonas gingivalis and Actinomyces naeslundii and promote the mineralization of the cementum. BMC Oral Health.

[25] Erdem A, Metzler D, Cha DK, Huang CP (2015). The short-term toxic effects of TiO _2_ nanoparticles toward bacteria through viability, cellular respiration, and lipid peroxidation. Environ Sci Pollut Res Int.

[26] Hou J, Wang L, Wang C, Zhang S, Liu H, Li S, Wang X (2019). Toxicity and mechanisms of action of titanium dioxide nanoparticles in living organisms. J Environ Sci (China).

[27] Song W, Ge S (2019). Application of Antimicrobial Nanoparticles in Dentistry. Molecules.

